# Outcomes by MIC Values for Patients Treated with Isavuconazole or Voriconazole for Invasive Aspergillosis in the Phase 3 SECURE and VITAL Trials

**DOI:** 10.1128/AAC.01634-18

**Published:** 2018-12-21

**Authors:** David R. Andes, Mahmoud A. Ghannoum, Pranab K. Mukherjee, Laura L. Kovanda, Qiaoyang Lu, Mark E. Jones, Anne Santerre Henriksen, Christopher Lademacher, William W. Hope

**Affiliations:** a University of Wisconsin—Madison, Madison, Wisconsin, USA; b Case Western Reserve University, Cleveland, Ohio, USA; c Astellas Pharma Global Development, Inc., Northbrook, Illinois, USA; d Basilea Pharmaceutica International Ltd., Basel, Switzerland; e University of Liverpool, Liverpool, United Kingdom

**Keywords:** MIC, clinical trial, isavuconazole, isavuconazonium sulfate, voriconazole

## Abstract

This pooled analysis evaluated the relationship of isavuconazole and voriconazole MICs of *Aspergillus* pathogens at baseline with all-cause mortality and clinical outcomes following treatment with either drug in the SECURE and VITAL trials. Isavuconazole and voriconazole may have had reduced efficacy against pathogens with drug MICs of ≥16 µg/ml, but there was no relationship with clinical outcomes in cases where the MIC was <16 µg/ml for either drug.

## INTRODUCTION

Invasive fungal diseases (IFD) are serious medical conditions (1–3). For example, mortality rates for invasive aspergillosis (IA) at 6 weeks are approximately 20% (4). Treatment guidelines from the Infectious Diseases Society of America and the European Conference on Infections in Leukemia recommend voriconazole as a first-line agent for IA (5–7). On the basis of the results of the SECURE and VITAL phase 3 trials (8, 9), isavuconazole was approved by the U.S. Food and Drug Administration for the treatment of adults with IA or invasive mucormycosis and by the European Medicines Agency for treatment of adults with IA or with mucormycosis when amphotericin B is not appropriate. Isavuconazole has also now been included in guidelines as an alternative first-line agent (5–7). Clinical breakpoints for isavuconazole have now been defined by the European Committee on Antimicrobial Susceptibility Testing (EUCAST) for several *Aspergillus* spp. (10).

This analysis describes the relationships of MICs of isavuconazole and voriconazole against *Aspergillus* pathogens at baseline in pooled data from the SECURE (8) and VITAL trials (11), which compared isavuconazole (200 mg [administered as 372 mg prodrug dose] orally or intravenously every 8 h for 48 h, then once daily) and voriconazole (6 mg/kg intravenously twice daily on day 1, 4 mg/kg intravenously twice daily on day 2, then intravenously 4 mg/kg twice daily or orally 200 mg twice daily from day 3 onwards) for the primary treatment of IFD caused by *Aspergillus* spp. and other filamentous fungi, and efficacy of isavuconazole for patients with IA and renal impairment or with other rare IFDs, respectively.

Antifungal susceptibilities were tested (Clinical and Laboratory Standards Institute [CLSI] and EUCAST methods) (12, 13) in 96 *Aspergillus* sp. isolates (morphologically and molecularly confirmed [14, 15]) obtained at baseline. For all pathogens from patients treated with isavuconazole (*n* = 55 patients; mean age [standard deviation {SD}] 49.5 [17.5] years; 71 *Aspergillus* samples), the CLSI and EUCAST MIC values were the same (MIC_50_, 1 µg/ml; MIC_90_, 4 µg/ml). Furthermore, the ranges of MICs were comparable (CLSI, 0.25 to 32 µg/ml; EUCAST, 0.12 to 32 µg/ml).

The MIC_50_ and MIC_90_ for pathogens from patients treated with voriconazole (*n* = 23 patients; mean age [SD] 53.1 [16.1] years; 25 *Aspergillus* samples) were 1 µg/ml and 2 µg/ml, respectively, as determined using either CLSI or EUCAST methods. The ranges of MICs were 0.25 to 2 (CLSI) and 0.25 to 16 (EUCAST) µg/ml. In summary, isavuconazole and voriconazole displayed potent *in vitro* activity against the majority of *Aspergillus* spp., with MICs consistent with those recently reported elsewhere (16–20).

**FIG 1:**
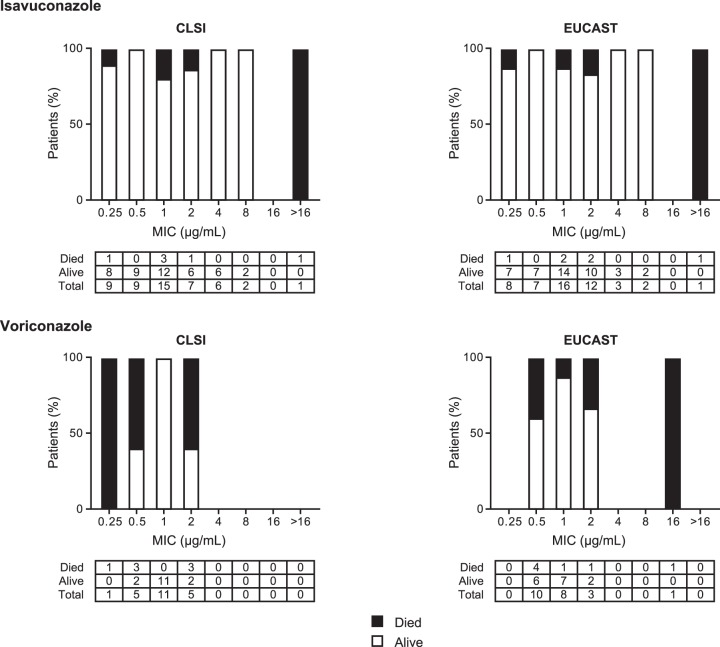
All-cause mortality in patients with *Aspergillus* spp. only treated with isavuconazole and voriconazole at day 42 using drug MICs for *Aspergillus* sp. isolates by CLSI and EUCAST methodologies. CLSI, Clinical and Laboratory Standards Institute; EUCAST, European Committee on Antimicrobial Susceptibility Testing.

All-cause mortality (ACM) through day 42 was assessed in all patients with proven, probable, or possible IFD who received at least one dose of study drug (Table 1; seen also Table S1 in ths supplemental material). Among isavuconazole-treated patients (*Aspergillus* spp. only), the ACM rates associated with pathogens having drug MICs of ≤1 µg/ml were 12.1% (4/33; CLSI) and 9.7% (3/31; EUCAST), whereas the ACM rates associated with pathogens having drug MICs of >1 µg/ml were 12.5% (2/16; CLSI) and 16.6% (3/18; EUCAST). Among voriconazole-treated patients (*Aspergillus* spp. only), the ACM rates associated with pathogens having drug MICs of ≤1 µg/ml were 23.5% (4/17; CLSI) and 27.8% (5/18; EUCAST), whereas the ACM rates associated with pathogens having drug MICs of >1 µg/ml were 60% (3/5; CLSI) and 50% (2/4; EUCAST). With the exception of one patient in the isavuconazole group who was infected with multiple fungal species, all patients in both treatment groups infected with *Aspergillus* spp. with drug MICs of ≥16 µg/ml (*n* = 2 for isavuconazole by either CLSI or EUCAST; *n* = 1 for voriconazole by EUCAST only) died by day 42. For patients with *Aspergillus*-only infections, ACM at day 42 was generally higher with voriconazole than with isavuconazole (Fig. 1) (Table 1; see also Table S1); however, the study was underpowered to enable a statistical comparison between the groups.

**TABLE 1:** All-cause mortality through day 42 classified by baseline drug MICs for *Aspergillus* sp. isolates alone or with other fungal pathogens: CLSI and EUCAST methodologiesa^*a*^

Methodology, treatment, and target	No. of isolates with indicated MIC (μg/ml)/total no. of isolates (% of total)^*b*^							
	0.25	0.5	1	2	4	8	16	>16
CLSI								
Isavuconazole								
*Aspergillus* spp. only	1/9 (11)	0/9	3/15 (20)	1/7 (14)	0/6	0/2		1/1 (100)
Multiple fungal spp.^*c*^	0/1			0/1		0/3	1/1 (100)	
Voriconazole								
*Aspergillus* spp. only	1/1 (100)	3/5 (60)	0/11	3/5 (60)				
Multiple fungal spp.^*d*^						0/1		

EUCAST								
Isavuconazole								
*Aspergillus* spp. only	1/8 (13)	0/7	2/16 (17)	2/12 (17)	0/3	0/2		1/1 (100)
Multiple fungal spp.^*c*^	0/1		0/1	0/1	0/1		1/1 (100)	0/1
Voriconazole								
*Aspergillus* spp. only		4/10 (40)	1/8 (13)	1/3 (33)			1/1 (100)	
Multiple fungal spp.^*d*^					0/1			

a See the supplemental materials for ACM data for individual and multiple *Aspergillus* spp. Only baseline samples are included in this summary. CLSI, Clinical and Laboratory Standards Institute; EUCAST, European Committee on Antimicrobial Susceptibility Testing; MIC, minimum inhibitory concentration.

b The denominator represents the number of patients whose isolates had that drug MIC (where patients had multiple isolates, the isolate with the highest baseline drug MIC was used); the numerator denotes the number of patients who died. The outcome for a patient whose last known survival status was determined before day 42 or was missing and whose last assessment day was before day 42 was treated as representing death.

c Data include *Lichtheimia corymbifera* (*n* = 2 patients), *Fonsecaea monophora*, *Chaetomium brasiliense*, and *Rhizopus oryzae*.

d Data include *Penicillium piceum*.

The overall response (composite of clinical, mycological, and radiological [where relevant; see Table S2] responses at end of treatment [EOT] in patients with proven or probable IFD, as assessed by blind-data review committee) (21) was also investigated. Among isavuconazole-treated patients infected with pathogens (*Aspergillus* spp. only) with drug MICs of ≤1 µg/ml, overall successful responses were observed in 45.5% (15/33; CLSI) and 45.2% (14/31; EUCAST), whereas in cases of pathogens with drug MICs of >1 µg/ml, overall successful responses were observed in 43.8% (7/16; CLSI) and 44.4% (8/18; EUCAST) (Table 2) (see also Fig. S1 and Table S3 in the supplemental material). Among voriconazole-treated patients infected with pathogens (*Aspergillus* spp. only) with drug MICs of ≤1 µg/ml, overall successful responses were observed in 47.1% (8/17; CLSI) and 44.4% (8/18; EUCAST), whereas the overall successful responses observed for those whose pathogens had drug MICs of >1 µg/ml were 20.0% (1/5; CLSI) and 25.0% (1/4; EUCAST).

**TABLE 2:** Overall, clinical, and mycological responses at EOT classified by baseline drug MICs for *Aspergillus* sp. isolates alone or with other fungal pathogens: CLSI and EUCAST methodologiesa^*a*^

Methodology, treatment, and target	Outcome	No. of isolates with indicated MIC (μg/ml)/total no. of isolates (% of total)^*b*^							
		0.25	0.5	1	2	4	8	16	>16
CLSI									
Isavuconazole									
*Aspergillus* spp. only	Overall success	4/9 (44)	5/9 (56)	6/15 (40)	3/7 (43)	3/6 (50)	1/2 (50)		0/1
	Clinical success	6/9 (67)	9/9 (100)	9/15 (60)	5/7 (71)	4/6 (67)	2/2 (100)		0/1
	Mycological success	4/9 (44)	6/9 (44)	7/15 (47)	3/7 (43)	3/6 (50)	1/2 (50)		0/1
Multiple fungal spp.^*c*^	Overall success	0/1			0/1		1/3 (33)	0/1	
	Clinical success	1/1 (100)			0/1		2/3 (67)	0/1	
	Mycological success	1/1 (100)			0/1		1/3 (33)	0/1	
Voriconazole									
*Aspergillus* spp. only	Overall success	0/1	1/5 (20)	7/11 (64)	1/5 (20)				
	Clinical success	0/1	2/5 (40)	10/11 (91)	2/5 (40)				
	Mycological success	0/1	1/5 (20)	8/11 (73)	1/5 (20)				
Multiple fungal spp.^*d*^	Overall success						1/1 (100)		
	Clinical success						1/1 (100)		
	Mycological success						1/1 (100)		

EUCAST									
Isavuconazole									
*Aspergillus* spp. only	Overall success	4/8 (50)	4/7 (57)	6/16 (38)	5/12 (42)	1/3 (33)	2/2 (100)		0/1
	Clinical success	5/8 (63)	7/7 (100)	11/16 (69)	8/12 (67)	2/3 (67)	2/2 (100)		0/1
	Mycological success	4/8 (50)	4/7 (57)	8/16 (50)	5/12 (42)	1/3 (33)	2/2 (100)		0/1
Multiple fungal spp.^*c*^	Overall success	0/1		1/1 (100)	0/1	0/1		0/1	0/1
	Clinical success	1/1 (100)		1/1 (100)	0/1	1/1 (100)		0/1	0/1
	Mycological success	1/1 (100)		1/1 (100)	0/1	0/1		0/1	0/1
Voriconazole									
*Aspergillus* spp. only	Overall success		3/10 (30)	5/8 (63)	1/3 (33)			0/1	
	Clinical success		6/10 (60)	6/8 (75)	2/3 (67)			0/1	
	Mycological success		4/10 (40)	5/8 (63)	1/3 (33)			0/1	
Multiple fungal spp.^*d*^	Overall success					1/1 (100)			
	Clinical success					1/1 (100)			
	Mycological success					1/1 (100)			

a See the supplemental materials for responses for individual and multiple *Aspergillus* spp. CLSI, Clinical and Laboratory Standards Institute; EUCAST, European Committee on Antimicrobial Susceptibility Testing; EOT, end of treatment.

b The denominator represents the number of patients whose isolates had that drug MIC (where patients had multiple isolates, the isolate with the highest baseline drug MIC was used); the numerator denotes the number of patients who demonstrated a response of success.

c Data include *Lichtheimia corymbifera* (*n* = 2 patients), *Fonsecaea monophora*, *Chaetomium brasiliense*, and *Rhizopus oryzae*.

d Data include *Penicillium piceum*.

These analyses of primarily wild-type isolates from the SECURE and VITAL trials suggest that a clearly defined relationship between *in vitro* susceptibility and clinical outcomes may not be detectable below the epidemiological cutoff value of ≤1 µg/ml (CLSI) (22) or ≤2 µg/ml (EUCAST) for most common *Aspergillus* spp. (23). Moreover, this analysis was limited by the small number of isolates, which prevented statistical analysis of the relationship between susceptibility and outcomes and precluded comments on suboptimal outcomes associated with high MICs. Using clinical data to verify the adequacy of current breakpoints would require much larger studies and enrollment of patients with infections by strains with drug MICs higher than the breakpoint, the latter of which would not be ethically tenable.

## Supplementary Material

Supplemental file 1
