# Supplementary material for: Outcomes by MIC Values for Patients Treated with Isavuconazole or Voriconazole for Invasive Aspergillosis in the Phase 3 SECURE and VITAL Trials
Source: Antimicrob Agents Chemother. 2018 Dec 21;63(1):e01634-18. doi: 10.1128/AAC.01634-18 (PMC6325202; doi:10.1128/AAC.01634-18)
Supplement: Supplemental file 1 [file 86e917de5d07eeeaa62166d6d8a45784_AAC.01634-18-s0001.pdf]

TABLE S1 All-cause mortality through Day 42 by baseline MICs for individual and multiple *Aspergillus* spp. isolates: CLSI and EUCAST methodologies

	MICs (µg/mL)[a]							
	0.25	0.5	1	2	4	8	16	>16
**CLSI**								
Isavuconazole								
A. flavus	-	0/1	1/6 (17%)	0/2	0/1	-	-	-
A. fumigatus	0/7	0/6	2/8 (25%)	0/3	-	-	-	1/1 (100%)
A. niger	-	-	-	1/1 (100%)	0/4	0/2	-	-
A. terreus	0	0	0/1	0	0/1	-	-	-
Multiple *Aspergillus* spp.	1/2 (50%)	0/2	-	0/1	-	-	-	-
Voriconazole								
A. flavus	-	0/1	0/3	1/2 (50%)	-	-	-	-
A. fumigatus	1/1 (100%)	3/4 (75%)	0/7	2/3 (67%)	-	-	-	-
A. terreus	-	-	0/1	-	-	-	-	-

**EUCAST**								
Isavuconazole								
A. flavus	0/1	-	0/3	1/6 (17%)	-	-	-	-
A. fumigatus	0/5	0/6	2/11 (18%)	0/2	-	-	-	1/1 (100%)
A. niger	-	-	-	1/2 (50%)	0/3	0/2	-	-
A. terreus	-	0/1	-	0/1	-	-	-	-
Multiple *Aspergillus* spp.	1/2 (50%)	-	0/2	0/1	-	-	-	-
Voriconazole								
A. flavus	-	-	0/5	1/1 (100%)	-	-	-	-
A. fumigatus	-	4/9 (44%)	1/3 (33%)	0/2	-	-	1/1 (100%)	-
A. terreus	-	0/1	-	-	-	-	-	-

[a]The denominator is the number of patients whose isolates had that MIC (where patients had multiple isolates, the isolate with the highest baseline MIC was used); the numerator denotes the number of patients who died. A patient with the last known survival status was before Day 42 or missing and the last assessment day was before Day 42 was treated as death.

Only baseline samples are included in this summary.

CLSI, Clinical and Laboratory Standards Institute; EUCAST, European Committee on Antimicrobial Susceptibility Testing; MIC, minimum inhibitory concentration.

Table S2 DRC-Assessment definitions of clinical, mycological, and radiological responses in the SECURE and VITAL trials

**Outcome**	**Clinical response**
Success	Complete or partial resolution of attributable clinical symptoms and physical findings
Failure	No resolution of any attributable clinical symptoms and physical findings and/or worsening
Not applicable	No attributable signs and symptoms present at baseline and no symptoms attributable to invasive fungal disease developed post baseline
**Outcome**	**Mycological response**
Success	Documented or presumed eradication
Failure	Persistence or presumed persistence
Not applicable	No mycological evidence available at baseline
**Time point**	**Radiological response**
Day 42	• Success (improvement of at least 25% from baseline)<br>• Failure<br>• No post-baseline radiology available for patient with baseline evidence of radiological disease<br>• Radiology not applicable at baseline

Day 84	• Success (improvement of at least 50% from baseline)<br>• Failure<br>• No post-baseline radiology available for patient with baseline evidence of radiological disease<br>• Radiology not applicable at baseline
EOT	• Success (improvement of at least 25% from baseline [if EOT occurred prior to day 42] or at least 50% from baseline [if EOT occurred after day 42])<br>• Failure<br>• No post-baseline radiology available for patient with baseline evidence of radiological disease<br>• Radiology not applicable at baseline

DRC, data review committee; EOT, end of treatment.

TABLE S3 Overall, clinical, and mycological response at EOT by baseline MICs for individual and multiple *Aspergillus* spp. isolates: CLSI and EUCAST methodologies

	Outcome	MICs (µg/mL)[a]							
		0.25	0.5	1	2	4	8	16	>16
**CLSI**									
Isavuconazole									
A. flavus	Overall success	-	1/1 (100%)	4/6 (67%)	1/2 (50%)	0/1	-	-	-
	Clinical success	-	1/1 (100%)	5/6 (83%)	1/2 (50%)	1/1 (100%)	-	-	-
	Mycological success	-	1/1 (100%)	4/6 (67%)	1/2 (50%)	0/1	-	-	-
A. fumigatus	Overall success	3/7 (43%)	3/6 (50%)	2/8 (25%)	2/3 (67%)	-	-	-	0/1
	Clinical success	5/7 (71%)	6/6 (100%)	3/8 (38%)	3/3 (100%)	-	-	-	0/1
	Mycological success	3/7 (43%)	4/5 (67%)	3/8 (38%)	2/3 (67%)	-	-	-	0/1
A. niger	Overall success	-	-	-	0/1	3/4 (75%)	1/2 (50%)	-	-
	Clinical success	-	-	-	0/1	3/4 (75%)	2/2 (100%)	-	-

	Mycological success	-	-	-	0/1	3/4 (75%)	1/2 (50%)	-	-
A. terreus	Overall success	-	-	0/1	-	0/1	-	-	-
	Clinical success	-	-	1/1 (100%)	-	0/1	5-	-	-
	Mycological success	-	-	0/1	-	0/1	-	-	-
Multiple *Aspergillus* spp.	Overall success	1/2 (50%)	1/2 (50%)	-	0/1	-	-	-	-
	Clinical success	1/2 (50%)	2/2 (100%)	-	1/1 (100%)	-	-	-	-
	Mycological success	1/2 (50%)	1/2 (50%)	-	0/1	-	-	-	-
Voriconazole									
A. flavus	Overall success	-	0/1	3/3 (100%)	1/2 (50%)	-	-	-	-
	Clinical success	-	1/1 (100%)	2/3 (67%)	1/2 (50%)	-	-	-	-
	Mycological success	-	0/1	3/3 (100%)	1/2 (50%)	-	-	-	-

A. fumigatus	Overall success	0/1	1/4 (25%)	4/7 (57%)	0/3	-	-	-	-
	Clinical success	0/1	1/4 (25%)	7/7 (100%)	3/3 (100%)	-	-	-	-
	Mycological success	0/1	1/4 (25%)	5/7 (71%)	0/3	-	-	-	-
A. terreus	Overall success	-	-	0/1	-	-	-	-	-
	Clinical success	-	-	1/1 (100%)	-	-	-	-	-
	Mycological success	-	-	0/1	-	-	-	-	-
**EUCAST**									
Isavuconazole									
A. flavus	Overall success	1/1 (100%)	-	2/3 (67%)	3/6 (50%)	-	-	-	-
	Clinical success	1/1 (100%)	-	3/3 (100%)	4/6 (67%)	-	-	-	-
	Mycological success	1/1 (100%)	-	2/3 (67%)	3/6 (50%)	-	-	-	-
A. fumigatus	Overall success	2/5 (40%)	4/6 (67%)	3/11 (27%)	1/2 (50%)	-	-	-	-
	Clinical success	3/5 (60%)	6/6 (100%)	6/11 (55%)	2/2 (100%)	-	-	-	-

	Mycological success	2/5 (40%)	4/6 (67%)	5/11 (45%)	1/2 (50%)	-	-	-	-
A. niger	Overall success	-	-	-	1/2 (50%)	1/3 (33%)	2/2 (100%)	-	-
	Clinical success	-	-	-	1/2 (50%)	2/3 (67%)	2/2 (100%)	-	-
	Mycological success	-	-	-	1/2 (50%)	1/3 (33%)	2/2 (100%)	-	-
A. terreus	Overall success	-	0/1	-	0/1		-	-	-
	Clinical success	-	1/1 (100%)	-	0/1		-	-	-
	Mycological success	-	0/1	-	0/1		-	-	-
Multiple *Aspergillus* spp.	Overall success	1/2 (50%)	-	1/2 (50%)	0/1	-	-	-	-
	Clinical success	1/2 (50%)	-	2/2 (100%)	1/1 (100%)	-	-	-	-
	Mycological success	1/2 (50%)	-	1/2 (50%)	0/1	-	-	-	-
Voriconazole									

A. flavus	Overall success	-	-	4/5 (100%)	0/1	-	-	-	-
	Clinical success	-	-	4/5 (100%)	0/1	-	-	-	-
	Mycological success	-	-	4/5 (100%)	0/1	-	-	-	-
A. fumigatus	Overall success	-	3/9 (33%)	1/3 (33%)	1/2 (50%)	-	-	0/1	-
	Clinical success	-	5/9 (56%)	2/3 (67%)	2/2 (100%)	-	-	0/1	-
	Mycological success	-	4/9 (44%)	1/3 (33%)	1/2 (50%)	-	-	0/1	-
A. terreus	Overall success	-	0/1	-	-	-	-	-	-
	Clinical success	-	1/1 (100%)	-	-	-	-	-	-
	Mycological success	-	0/1	-	-	-	-	-	-

[a]The denominator is the number of patients whose isolates had that MIC (where patients had multiple isolates, the isolate with the highest baseline MIC was used); the numerator denotes the number of patients who demonstrated a response of success.

CLSI, Clinical and Laboratory Standards Institute; EOT, end of treatment; MIC, minimum inhibitory concentration; EOT, end of treatment; EUCAST, European Committee on Antimicrobial Susceptibility Testing; MIC, minimum inhibitory concentration.

FIG S1 Overall, clinical, and mycological response in patients with *Aspergillus* spp. only at EOT by MICs for *Aspergillus* spp. isolates by CLSI methodologies.

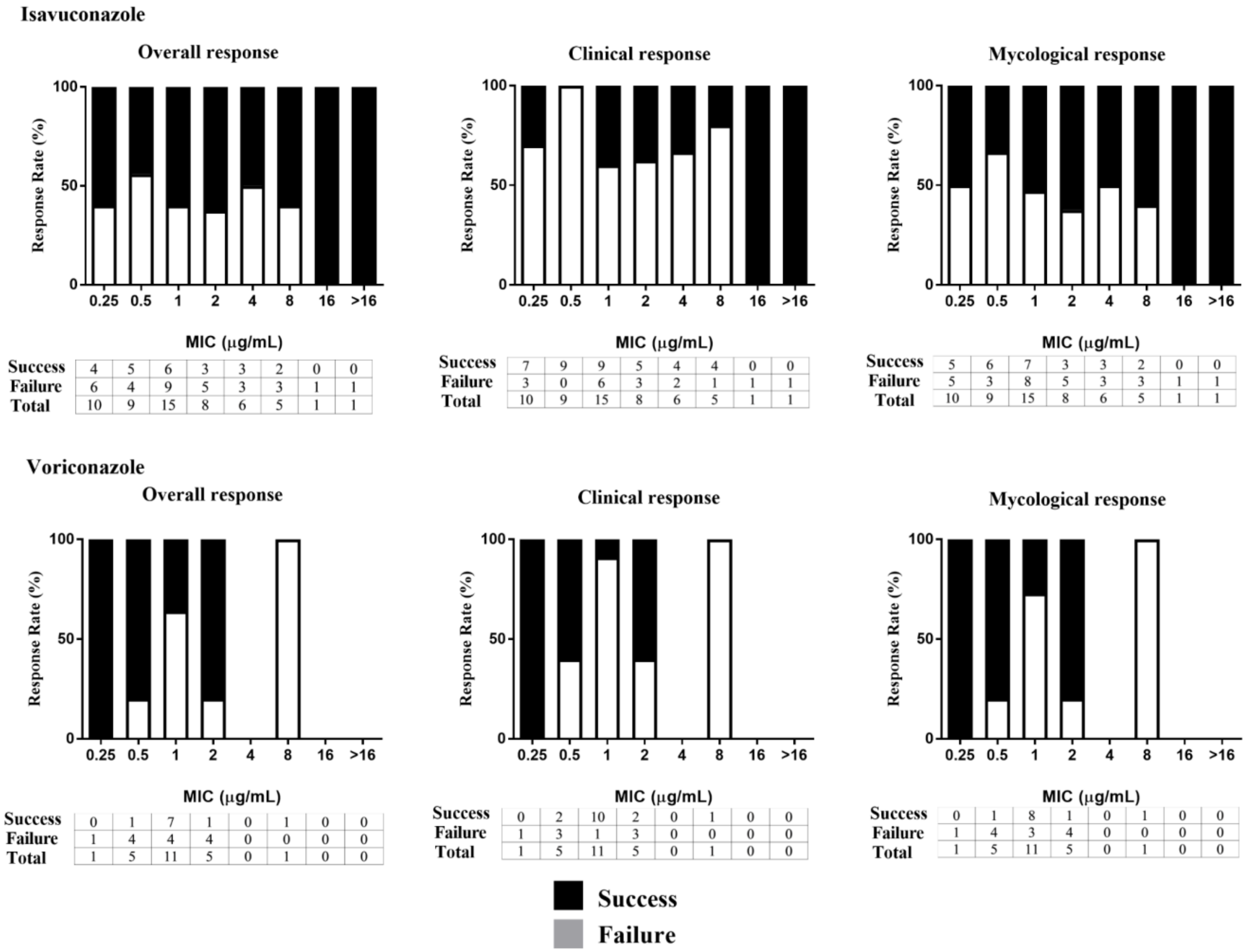

Isavuconazole – Overall response

MIC (μg/mL)	0.25	0.5	1	2	4	8	16	>16
Success	4	5	6	3	3	2	0	0
Failure	6	4	9	5	3	3	1	1
Total	10	9	15	8	6	5	1	1

Isavuconazole – Clinical response

MIC (μg/mL)	0.25	0.5	1	2	4	8	16	>16
Success	7	9	9	5	4	4	0	0
Failure	3	0	6	3	2	1	1	1
Total	10	9	15	8	6	5	1	1

Isavuconazole – Mycological response

MIC (μg/mL)	0.25	0.5	1	2	4	8	16	>16
Success	5	6	7	3	3	2	0	0
Failure	5	3	8	5	3	3	1	1
Total	10	9	15	8	6	5	1	1

Voriconazole – Overall response

MIC (μg/mL)	0.25	0.5	1	2	4	8	16	>16
Success	0	1	7	1	0	1	0	0
Failure	1	4	4	4	0	0	0	0
Total	1	5	11	5	0	1	0	0

Voriconazole – Clinical response

MIC (μg/mL)	0.25	0.5	1	2	4	8	16	>16
Success	0	2	10	2	0	1	0	0
Failure	1	3	1	3	0	0	0	0
Total	1	5	11	5	0	1	0	0

Voriconazole – Mycological response

MIC (μg/mL)	0.25	0.5	1	2	4	8	16	>16
Success	0	1	8	1	0	1	0	0
Failure	1	4	3	4	0	0	0	0
Total	1	5	11	5	0	1	0	0

Baseline isolates from patients in the isavuconazole arm were tested with isavuconazole; baseline isolates from patients in the voriconazole arm were tested with voriconazole; where patients had multiple isolates, the isolate with the highest baseline MIC was used.

CLSI, Clinical and Laboratory Standards Institute; EOT, end of treatment; MIC, minimum inhibitory concentration.
